# Predicting suicide attempts in a high-risk clinical cohort of adolescents using machine-learning

**DOI:** 10.1007/s00787-026-02963-2

**Published:** 2026-01-31

**Authors:** Christian Hertel, Noel Strahm, Stefan Lerch, Corinna Reichl, Julian Koenig, Marialuisa Cavelti, Michael Kaess

**Affiliations:** 1 University Hospital of Child and Adolescent Psychiatry and Psychotherapy, University of Bern, Bolligenstrasse 111, 3000, Bern 60, Switzerland; 2 Faculty of Medicine, Department of Child and Adolescent Psychiatry, Psychosomatics and Psychotherapy, University of Cologne, University Hospital Cologne, Cologne, Germany; 3 Department of Child and Adolescent Psychiatry, Centre for Psychosocial Medicine, University Hospital Heidelberg, Heidelberg, Germany

**Keywords:** Suicide attempt, Adolescent, Machine learning, Prediction, Clinical cohort

## Abstract

While suicide prevention is a major global health priority in youth, it remains challenging to identify those at risk for future suicidal behavior. This study aimed to investigate the potential of machine learning (ML) based approaches to predict suicide attempts (SA) among high-risk adolescents. We applied three ML-algorithms – elastic net, random forest and extreme gradient boosting – to longitudinal clinical data, and compared their performance to a traditional statistical approach – logistic regression (LR). Models were trained using patient data from a clinical cohort (*N* = 255) of adolescents with self-harming and risk-taking behaviors. Forty-four predictor variables including sociodemographic information, features of suicidal thoughts and behaviors, psychiatric disorders, global functioning scores and adverse childhood events were obtained at baseline and selected to predict future SAs within a two-year follow-up period. Performance metrics included area under the curve (AUC), Brier score, sensitivity, specificity, positive predictive value (PPV), and negative predictive value (NPV). Moreover, individual predictor importance was explored. ML-algorithms outperformed LR in terms of overall predictive accuracy (AUC = 0.75–0.79 vs. 0.72) and model calibration (Brier scores = 0.18–0.20 vs. 0.24). A prior SA stood out as the most important predictor variable across all algorithms. Our findings demonstrate that SA can be predicted with good overall accuracy, even among patients with high prevalence of suicidal behaviors. The clinical utility of ML-based SA-predictions is subject to several limitations (e.g., ethical, legal, clinical). Alongside improvements of predictive performance, future research needs to address clinical and ethical implications of ML-based risk detection.

**Supplementary Information:**

The online version contains supplementary material available at 10.1007/s00787-026-02963-2.

## Introduction

Suicide is one of the leading causes of death among adolescents worldwide, and suicide attempts (SA) occur in 5–10% of the adolescent population [1]. One way to reduce the risk of future SA is to identify individuals who are at high-risk for suicidal behavior and tailor specific interventions to them [2].

Over the past five decades, numerous risk factors associated with suicidal behavior have been uncovered [3]; however, current evidence suggests that suicide risk prediction, whether based on clinical judgment or structured risk assessment tools, remains ineffective [4]. This is primarily due to the continued inability to accurately identify individuals at risk of suicidal behavior, with predictive accuracy only marginally exceeding chance [3]. Some scholars argue that suicide, by its very nature, is inherently unpredictable [4]. Others criticize existing prediction models for focusing predominantly on static risk factors rather than incorporating dynamic factors, which often results in models that fail to offer actionable insights for clinical decision-making [5]. As a consequence, clinical guidelines [6] have shifted from an emphasis on prediction to the active management of suicide risk.

While there is justified scepticism regarding the prospective prediction of suicide or SA based on static trait markers, there may still be significant value in improving risk stratification within at-risk populations. Such an approach could facilitate more tailored treatments, and included risk management strategies, suited to individual needs. Thus, the prediction of suicide and SA remains a critical area of research [7]. Given that suicidal behavior is likely influenced by a complex interplay of various risk factors, predictive accuracy could be improved if models were to allow complex combinations of a large number of potential risk factors. However, the field has traditionally relied on conventional statistical methods, which often impose constraints on the number of predictors and struggle to capture intricate non-linear associations between them [3, 7].

A shift towards machine-based learning (ML) has helped to address these limitations and predictive accuracy has improved in recent years [8]. In adolescents, most research has been conducted in large community samples showing acceptable to good predictions above chance level (e.g [9]). When comparing ML to conventional statistical models, results have been mixed with some studies showing superior predictions in ML models (e.g [10, 11]), while others did not (e.g [12, 13]). Regarding clinical samples in adolescents, only few studies have used ML-approaches [11, 14–17], showing promising accuracy in the prediction of SAs but also suffering from several limitations (sample size, unavailable data on type and date of SA, overfitting, different algorithms).

Regarding the most important predictors in adolescent SAs identified within the models, there seems to be some consistency: history of depression or substance abuse, medication, and prior SA were reported in several studies [11, 14, 16, 17] with the latter consistently identified as the strongest risk factor. Yet, there was no clear consistency regarding the types of ML algorithms used (e.g. Random Forest, LASSO, Elastic Net, Natural Language Processing) [11, 14, 16, 17].

Especially larger studies were either cross-sectional and/or used electronic health record (EHR)-data which tend to include variables lacking relevance for clinical decision making. Overall, reported area under the curve (AUC) generally was classified as “good” (around 0.8) but both community samples as well as EHR cohorts are often associated with low base rates of suicidal behavior. Predicting SAs within a specific high-risk population could improve clinical relevance and facilitate the translation of research insights into guidelines for clinical practice [18]. To further develop this area of research, we try to combine the strengths of past research and cover issues that have scarcely been addressed. This paper focusses on a highly vulnerable subgroup of clinically referred adolescents with high prevalence of self-harm and risk-taking behaviors, using a broad range of clinical and sociodemographic predictor variables, a large sample size and a longitudinal design with a two-year follow-up period. To make our results comparable, we used several ML-algorithms that had been used in other studies. To the best of our knowledge, this specific high-risk population has not been studied with this methodological approach before.

The aim of our study was to assess the potential of ML-based strategies using structured clinical data within a clinical cohort of high-risk adolescents. We (1) hypothesized that SA-predictions would be more accurate for ML-algorithms compared to traditional logistic regression and (2) explored which predictor variables were most important when predicting future SAs.

## Methods

### Participants and procedure

Patient data was obtained from a specialized outpatient clinic for adolescents (aged 12 to 17 years) (AtR!Sk; University Hospital Heidelberg, Germany) [19] with high rates of repetitive non-suicidal self-injury (NSSI), risk-taking behaviour (e.g. substance use) as well as previous suicide attempts, alongside with clinical diagnoses of major depression and borderline personality disorder symptoms within this cohort [20, 21]. Consistent with other research [22], we consider this a high-risk sample for subsequent suicidal behavior. The study was approved by the Ethical Committee of the Medical Faculty, Heidelberg University, Germany (Study: ID S-449/2013) and was performed in line with the principles of the Declaration of Helsinki. All patients, as well as their legal guardians, provided written informed consent. Within the study, patients completed diagnostic assessments at baseline (T0), and again at 12 (T1) and 24 months (T2) follow-ups (baseline assessments between 2013 and 2020). For the present study, we included all patients from the AtR!Sk cohort aged 12–17 years, who completed diagnostic assessments at baseline (T0) and provided information on suicidal behaviors at the two year follow-up (T2). Exclusion criteria at baseline were insufficient proficiency in the German language; intellectual impairments; acute psychotic disorder; and/or the acute intention to commit suicide or cause harm to others; and a diagnosis of bipolar disorder, schizophrenia, or schizoaffective disorder. The final study sample consisted of *n* = 255 patients. Patients were either classified as SA-positive – patients who had made at least one SA during the two years following their baseline assessment, or SA-negative – patients who did not report a SA during the same two-year period.

### Assessments

A total of forty-four potentially predictive variables from structured clinical assessments were selected to ensure that different risk factor categories were covered, and to account for the possibility of intricate interactions among a large number of risk factors [3]. The following broad categories were taken into account: socio demographics, self-injurious thoughts and behaviors interview (SITBI-G) [23] measures, clinical measures, global clinical impressions, and adverse experiences. Some of the SITBI-variables are nested due to overlapping time frames (weeks, months, lifetime). All predictors were obtained at baseline. A detailed overview of all predictors is shown in the online supplementary material (STable 1, SFigure 1).

### Missing data

Missing data was imputed via multiple imputation by chained equations [24], using the *mice* package [25] in R [26]. For further details, see online supplementary material (SFigure 2).

### Statistical analysis

Predictions were based on three ML-algorithms - elastic net penalized logistic regression (EN) [27], random forest (RF) [28] and extreme gradient boosting (XGB) [29]. These ML-algorithms were selected due to their efficacy in similar contexts [13, 30] and to allow both non-linear and linear relations between predictor variables. To compare ML-algorithms to a traditional statistical approach, non-regularized logistic regression (LR) was used. A set of performance metrics was obtained from each model, including AUC, Brier score, sensitivity, specificity, positive predictive value (PPV) and negative predictive value (NPV). Details on the implementation of ML-algorithms, software packages, performance metrics and predictor importance are included in the online supplementary material.

## Results

Of the 255 adolescents included in the data analysis 224 (88%) were female, with an average age of 14.96 years (SD = 1.47). The majority of patients reported a history of self-harm thoughts and behaviors, including NSSI (*n* = 236; 93%), suicidal thoughts (*n* = 225; 88%), suicide planning (*n* = 136; 54%) and prior SA (*n* = 127; 50%). Among all patients, ninety-six individuals (*n* = 96; 38%) were classified as SA-positive. The remaining one hundred fifty-nine patients (*n* = 159; 62%) were classified as SA-negative. Approximately half of the patients lived with both parents (*n* = 114, 45%) and attended a Gymnasium (academic secondary school in Germany; *n* = 121, 48%). They reported high functional impairment (GAF Mean = 50 SD = 12) and elevated rates of adverse childhood experiences (sexual abuse *n* = 54, 22%; physical abuse *n* = 60, 25%; neglect *n* = 101, 41%). Details on baseline patient characteristics are included in the online supplementary material (STable 2).

### Model performance

Table 1 shows aggregated accuracy measures for all algorithms.

**Table 1:** Model performance

Accuracy Measures	LR [95% CI]	RF [95% CI]	XGB [95% CI]	EN [95% CI]
Sensitivity	0.56 [0.50, 0.62]	0.50 [0.44, 0.56]	0.53 [0.48, 0.58]	0.56 [0.51, 0.62]
Specificity	0.76 [0.73, 0.78]	0.82 [0.79, 0.84]	0.81 [0.79, 0.83]	0.82 [0.80, 0.84]
PPV	0.59 [0.55, 0.63]	0.63 [0.58, 0.67]	0.63 [0.59, 0.68]	0.67 [0.62, 0.71]
NPV	0.74 [0.72, 0.76]	0.73 [0.72, 0.75]	0.74 [0.72, 0.76]	0.76 [0.74, 0.78]
AUC	0.72 [0.70, 0.75]	0.75 [0.73, 0.78]	0.76 [0.73, 0.78]	0.79 [0.77, 0.81]
Brier score	0.24 [0.22, 0.25]	0.20 [0.19, 0.20]	0.20 [0.19, 0.20]	0.18 [0.18, 0.19]

*CI* confidence interval, *LR* logistic regression, *RF* random forest, *XGB* extreme gradient boosting, *EN* elastic net, *PPV* positive predictive value, *NPV* negative predictive value, *AUC* area under the receiver operator characteristic curve

**Fig. 1:**
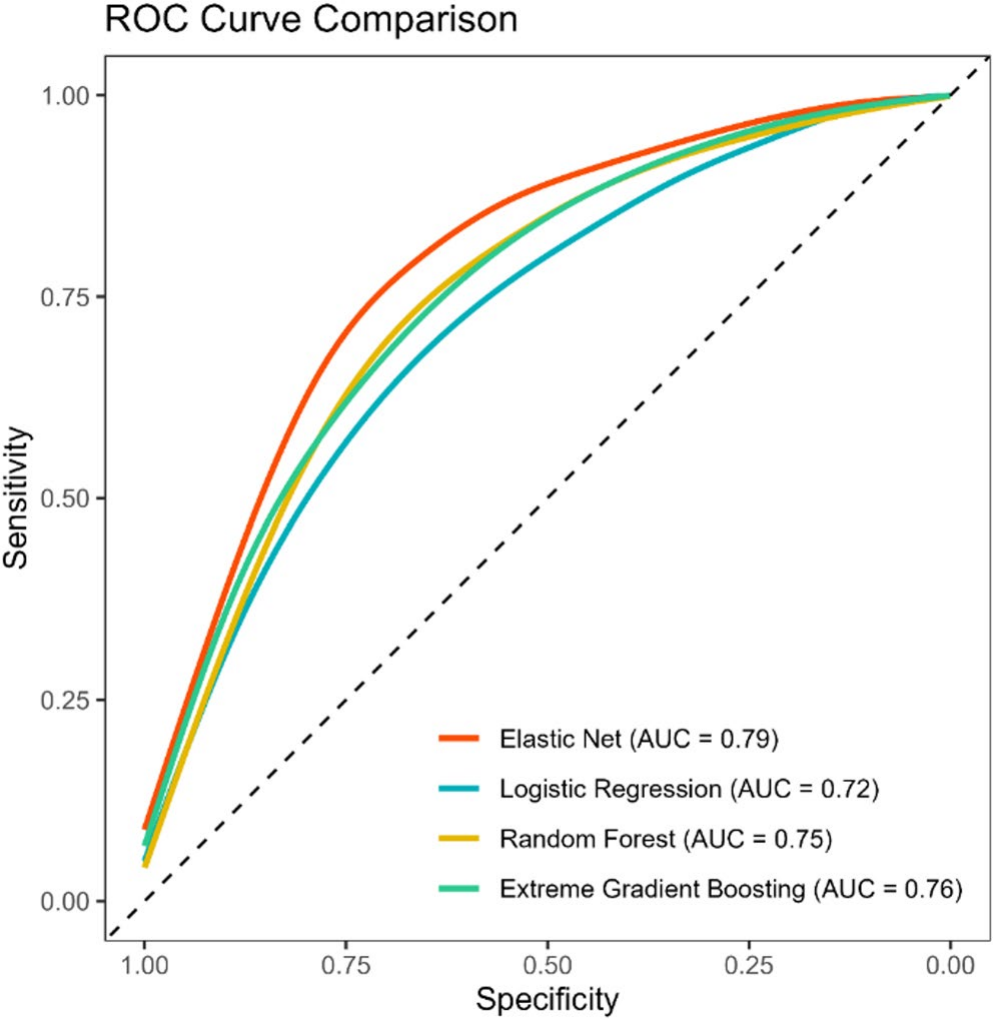
Comparison of AUC between algorithms for the prediction of suicide attempts. *Abbreviations*: AUC, area under the receiver operator characteristic curve. ROC, receiver operator characteristic

Overall, all algorithms showed good discriminative ability (Fig. 1, SFigure 3; further details including complete case analysis see online supplementary material, STable 3).

ML-algorithms showed slightly better overall predictive accuracy (AUCs = 0.75–0.79) and model calibration (Brier scores = 0.18–0.20), compared to LR (AUC = 0.72; Brier score = 0.24). EN exhibited the best discriminative performance across all accuracy metrics. In contrast, LR had the lowest values in terms of specificity, PPV and AUC, as well as the highest Brier score. At the predicted probability cutoff of 0.5, models were generally better at detecting SA-negative patients compared to SA-positive patients with specificity (0.76–0.82) and NPC (0.73–0.76) exceeding sensitivity (0.50–0.56) and PPV (0.59–0.67) across all algorithms. A summary of selected tuning parameters is shown in STable 4.

### Predictor importance

**Fig. 2:**
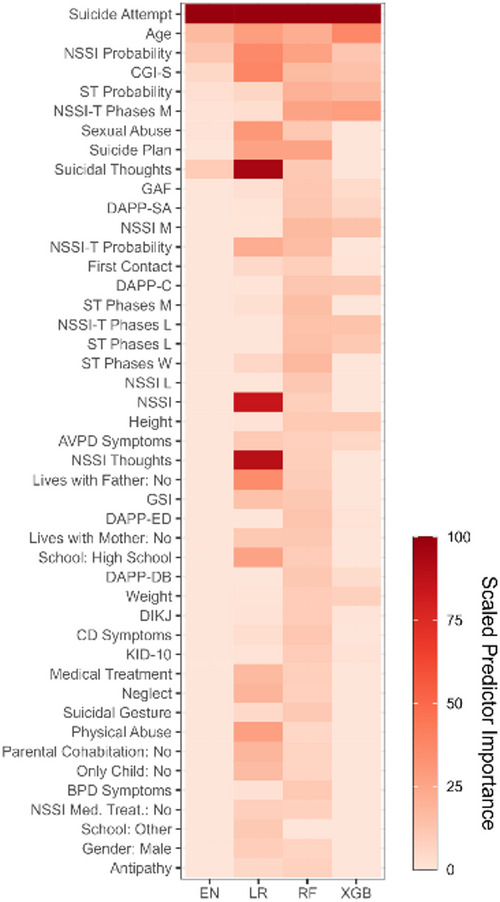
Predictor importance *Note.* Predictors are ordered by their median importance rank. Darker hue indicates higher importance (100 = most important, 0 = least important). *Abbreviations*: LR, logistic regression. RF, random forest. XGB, extreme gradient boosting. EN, elastic net. NSSI probability, self-rated probability of future non-suicidal self-injury. CGI-S, clinical global impression – severity. ST Probability, self-rated probability of future suicidal thoughts. NSSI-T Phases M, number of days with thoughts of non-suicidal self-injury in the past month. GAF, Global assessment of functioning. DAPP-SA, dimensional assessment of personality pathology social avoidance. NSSI M, number of days with non-suicidal self-injury in the past month. NSSI-T probability, self-rated probability of future thoughts of non-suicidal self-injury. First contact, year of the first contact to any psychiatric services. DAPP-C, dimensional assessment of personality pathology compulsiveness. ST phases M, number of days with suicidal thoughts in the past month. NSSI-T phases L, number of days with thoughts of non-suicidal self-injury lifetime. ST phases L, number of days with suicidal thoughts lifetime. ST phases L, number of days with suicidal thoughts lifetime. ST phases W, number of days with suicidal thoughts past week. NSSI L, number days with non-suicidal self-injury lifetime. NSSI, any non-suicidal self-injury lifetime. AVPD, number of symptoms of avoidant personality disorder. NSSI Thoughts, any thoughts of non-suicidal self-injury in lifetime. GSI, global severity index of symptoms. DAPP-ED, dimensional assessment of personality pathology emotional dysregulation. DAPP-DB, dimensional assessment of personality pathology dissocial behavior. DIKJ, total score symptoms of depression. CD symptoms, symptoms of conduct disorder. KID10, quality of life. Medical treatment, any current medication. Suicidal gesture, any suicidal gesture lifetime. BPD Symptoms, borderline personality symptoms. NSSI med. treat.: no, never received medical treatment because of non-suicidal self-injury. School: other, currently any other school than secondary or high school. Antipathy, antipathy by at least one parent

Predictor importance for each algorithm is shown in Fig. 2 (for details, see online supplementary material STable 5).

A previous SA ranked as the most important predictor across all algorithms. Age, the self-rated probability of future NSSI, as well as the CGI-S were among the most important predictors for all algorithms.

## Discussion

ML-based strategies have emerged as a promising approach for early detection of suicidal behavior among adolescents. Recent studies demonstrated good to very good accuracy when predicting SAs [9, 31–33], overcoming the fields longstanding struggle to surpass chance-level accuracy [3], though mostly based on community samples and EHR-data. In an effort to improve current insights, we applied ML to longitudinal structured clinical data from a clinical cohort of adolescents at high-risk for SAs. Our findings show that (1) ML-algorithms outperformed traditional LR in terms of overall predictive accuracy (AUCs = 0.75–0.79 vs. 0.72) and model calibration (Brier scores = 0.18–0.2 vs. 0.24). Additionally (2), an explorative analysis showed that a prior SA emerged as the most important variable when predicting future SAs, followed by age, self-rated probability of future NSSI and general symptom severity.

In line with other findings in clinical samples of adolescents [11, 14–17] our models performed similarly well with AUCs between 0.72 and 0.79. AUC is often the metric of choice in clinical prediction modelling, as it provides a general summary of a model’s overall ability to distinguish between different classes of patients. Which metric to choose when evaluating prediction models heavily depends on the context and should be based on a well-balanced trade of between sensitivity and specificity [34]. In this line, some critics argue that the clinical utility of SA-predictions relies more heavily on a model’s PPV [8, 35]. High PPVs suggest that when a SA is predicted, the prediction is likely to be accurate, minimizing the number of false positives. Despite high AUCs, most ML-based SA-prediction models are currently limited by low PPVs. One study [8] compared PPVs across several studies, concluding that most models produced extremely low PPVs when predicting suicide mortality (PPVs ≤ 0.01), whilst PPVs varied heavily when predicting SAs (PPVs = 0.00–0.78). Our models showed comparatively high PPVs (0.59–0.67), indicating that when a SA was predicted, the prediction was correct in the majority of cases – an essential requirement for clinical utility. However, PPVs are elevated with higher prevalence of an outcome [36], making them highly dependent on the population of interest. In our study population, the rate of a future SA was high (38%), which may have contributed to high PPVs. Adding to that, we argue that evaluating prediction models in clinical samples should aim at minimizing false negatives (i.e., predicting no SA when in fact a SA occurs) as the prevalence of SAs in clinical samples is much higher than in community samples, making false negatives a less acceptable error. NPVs quantify the ability of the model to correctly identify true negatives and are hardly reported in other studies. NVPs in our models (0.73–0.76) range in the middle of other studies on adolescent patients, with some showing lower (< 0.70) [14, 15] and others showing higher NPVs [16, 37].

Nonetheless, these findings highlight the potential of ML-based SA-prediction tools in high-risk clinical cohorts as (a) a future SA was predicted with good overall accuracy, despite suicidal behavior being prevalent among all patients, and (b) high SA-prevalence facilitates high PPVs, which improves clinical utility.

As hypothesized, predictive accuracy was better for ML-algorithms, compared to traditional LR. Although ML-algorithms outperformed LR in several studies [17, 18, 38], they did not in others [13, 30]. A recent review has concluded that there is no empirical evidence of a significant performance benefit of ML-algorithms over LR in clinical prediction modelling [39]. The efficacy of algorithms depends on the specific characteristics of the data they are applied to. Given the differences in sample characteristics, predictor variables, ML-algorithms, age groups and study designs, there is no algorithm universally considered the best for predicting SAs. This limits our ability to generalize our findings to other contexts, thereby posing another barrier in the process of bringing ML-based decision-making into clinical practice.

For specific predictor variables, there were some consistencies (i.e., age, self-rated future NSSI-probability, and CGI-S ranking high everywhere) and one clear constant between all algorithms: A previous SA emerged as the most important predictor variable. The authors would like to point out, that the model with the best performance (EN) weighs the most important predictors more strongly [40]. Consistent with previous findings in meta-analyses [3, 41] and other ML-studies [11, 16, 18], this reinforces the significance of the already well established SA-history as the most salient risk factor for future self-harm, highlighting that this finding is in line with existing evidence rather than a novel finding.

Age and clinical severity have been identified as important predictors in other ML-studies as well [11, 16, 18], stressing the importance of including non-suicide related predictors in prediction models [33]. Generally, predictor variables directly linked to self-harming thoughts and behaviors, - particularly self-rated future NSSI-probability - tended to have higher importance scores than more semantically remote predictor groups, such as socio-demographic data and general clinical measures. This also is in line with other ML-studies [11, 16, 18] linking suicidal ideation, NSSI and SAs in the framework of the third variable theory and Joiner’s Interpersonal Theory of Suicide [22].

Finding risk-factors for future SAs might help to identify important targets for preventive measures. This resembles a promising approach as primary preventive measures show some effects [42]. Unfortunately, nearly all existing secondary suicide prevention interventions (i.e., addressing individuals who already had a SA) for adolescents have so far produced nonsignificant treatment effects [43]. While clinical suicide prevention has put a focus on adult patients with a history of SAs [44], evidence-based suicide prevention programs for adolescents, who previously attempted suicide are still scarce [45]. Our findings reaffirm the status of past suicide-attempters as particularly vulnerable to future self-harm and highlight the potential of ML-based SA-predictions in high-risk clinical cohorts.

Even though identifying which predictor variables are most influential when predicting future SAs might provide valuable insights for clinical practitioners, the interpretation of predictor importance is not trivial. In the present study, predictor importance should only be interpreted in the context of a particular algorithm, given that a predictor’s “importance” was determined by metrics specific to each algorithm. Some of our variables were nested within different time frames. As a result, the model may attribute greater importance to the broader lifetime variable—simply because it captures the largest amount of variance—while assigning lower importance to the more recent variables, even if they are clinically meaningful. This effect does not bias the predictive performance of the model, but it does influence how importance values should be interpreted, emphasizing that importance reflects unique statistical contribution rather than absolute clinical relevance. On a more general level, the interpretation of predictor importance is further complicated because ML-algorithms might determine which predictors they consider important, based on intricate data-patterns that are no longer understandable by humans [13]. This problem is common in ML-domains, as the ability of ML-algorithms to identify complex data patterns often comes at the expense of reduced interpretability [46, 47]. This further jeopardizes our ability to draw clear clinical implications from our results, as the majority of predictor variables showed no stability in their importance score and rank between algorithms.

While the overall utility of suicide risk prediction remains a topic of debate among researchers and clinicians, we maintain that identifying patients at risk for suicide attempts (SA) is a critical step toward reducing these behaviors. Specifically, such identification can enable more effective risk stratification, thereby facilitating the implementation of targeted interventions and appropriate risk management strategies for those who need them most. This is especially important in the context of rising demands of care for youth self-harm and SA (e.g [48])., which is often coupled with limited resources for mental healthcare. However, there are still significant barriers to integrating machine learning (ML)-based decision-making into clinical practice. In addition to improving predictive accuracy, future research should increasingly focus on the clinical implications of suicide risk detection. Predicting risk is only valuable when it is followed by tailored interventions for those identified as being at risk [8, 49].

On a more general level, other challenges of implementing ML in clinical practice (e.g., implementation of which procedure in which setting, legal responsibility in case of clinical warning signs) as well as ethical dilemmas (e.g., consequences of real-time risk monitoring, bias of algorithms, communication of risk) have been addressed in detail [34]. Most crucial in the context of this paper is the potential of ML for not only improving the prediction of SAs but also its potential in tailoring interventions to specific needs of a patient as central in precision medicine/personalized treatment [5, 50]. The latter has hardly been addressed, and further research is needed to evaluate this important aspect of ML in the prevention of SAs.

## Strengths and limitations

First, predicting SAs among adolescents is relatively novel, as most studies have predominantly focused on adult populations [51]. Second, by investigating a high-risk clinical cohort, we showed that future SAs can be predicted with good accuracy, even in populations with high overlap in potentially predictive characteristics. Third, the majority of studies using ML to predict future SAs are cross-sectional in nature [7], whereas we used longitudinal data. Fourth, using data from a clinical cohort study enabled us to include variables that are valuable in clinical practice, which are typically less common in EHR data [34].

However, our study was also subject to several limitations: Larger samples routinely lead to more robust models [52], thus model-performance was potentially limited by the sample size of our study population. Due to the sample size, we did not perform external cross-validation, which is considered the gold standard to reduce the risk of overfitting. A future SA was predicted within a time frame of up to two years, however the exact time period between baseline and SA is unclear. This lack of information potentially hides more complex patterns, as predictive accuracy improves with shorter prediction windows, and predictor importance shifts across time [17, 38]. Our focus lied on distal risk factors, whereas future research should incorporate both distal and proximal risk factors. The use of ecological momentary assessment (EMA) and passive mobile sensing could help capture dynamic short-term changes of risk factors, as both suicidal ideation and its risk factors fluctuate considerably over short time periods [53]. Research on predictors of suicidal behavior has so far only insufficiently yielded results that can inform clinical decision-making and enhance patient safety [49]. EMA has the potential to bridge this gap by providing measurement-based care to both patients and clinicians, and by enabling adaptive just-in-time interventions tailored to patients’ specific needs using passive mobile sensing and wearables [54]. Given the dynamic and complex nature of the data, artificial intelligence and machine learning methods appear to be promising when combined with EMA [54]. Although EMA studies have begun to identify proximal risk factors (e.g., sleep, fluctuations in suicidal ideation, social conflicts), their potential to improve the management of suicidal patients is only beginning to be explored in current research [53].

The addition of other risk factor categories, such as biological factors or qualitative measures (texts such as search terms, messages written on smartphone or via social media) could also supply additional information, which might improve diagnostic accuracy. This is in line with some arguing for the use of natural language processing in ML-algorithms [55]. Lastly, we did not apply any dimensionality reduction techniques due to our rather atheoretical approach. Feature extraction and feature selection could improve model performance by removing irrelevant or redundant predictor variables [56].

## Conclusion

In conclusion, the present study demonstrates the potential of ML-based strategies in the development of suicide risk detection tools for at-risk adolescents and reaffirms that a prior SA is particularly important when predicting future SAs. Nonetheless, the clinical utility of ML-based SA-predictions remains limited. Predictions are not yet accurate enough to be clinically applicable, evidence-based suicide prevention treatments are lacking, and it is unclear whether ML-outcomes are generalizable and interpretable enough to justify clinical decision-making. While technical progress, such as improved accuracy of prediction-models is an essential requirement for clinical utility, both clinical and ethical implications of ML-based risk detection are crucial and must be addressed in future research.

## Supplementary Information

Below is the link to the electronic supplementary material.

Supplementary file 1 (DOCX 624 KB)

## Data Availability

The data that support the findings of this study are not openly available due to reasons of sensitivity and are available from the corresponding author upon reasonable request.
