# Supplementary material for: Predicting suicide attempts in a high-risk clinical cohort of adolescents using machine-learning
Source: Eur Child Adolesc Psychiatry. 2026 Jan 31;35(5):1565–73. doi: 10.1007/s00787-026-02963-2 (PMC13272205; doi:10.1007/s00787-026-02963-2)
Supplement: Supplementary file 1 — (DOCX 624 KB) [file 787_2026_2963_MOESM1_ESM.docx]

# Online Supplementary Material

**Methods**

**Predictors**

A detailed overview of all predictors is given in STable 1.

**STable 1** Detailed Predictor Overview

| Predictors | Description | Variable Type | Missing Rate | Imputation Method |
| --- | --- | --- | --- | --- |
|  |  |  |  |  |
| Socio-demographics |  |  |  |  |
| Age | Age in years | Continuous |  |  |
| Weight | Weight in kg | Continuous |  |  |
| Height | Height in cm | Continuous |  |  |
| Gender | Gender of patient | Factor [Female; Male] | 0% | - |
| Only Child | Is patient an only child? | Factor [Yes; No] | 0% | - |
| Parental Cohabitation | Do parents of patient live together? | Factor [Yes; No] | 0.39% | Logreg |
| Lives with Mother | Does patient live with their mother? | Factor [Yes; No] | 4.31% | Logreg |
| Lives with Father | Does patient live with their father? | Factor [Yes; No] | 13.33% | Logreg |
| School | Current School | Factor [Secondary School; High School; Other] | 0.39% | Polyreg |
|  |  |  |  |  |
| SITBI Measures |  |  |  |  |
| Suicidal Thoughts | Suicidal Thoughts in lifetime | Factor [Yes; No] | 0% | - |
| ST Phases L | Number of ST phases in lifetime | Continuous | 0.78% | Pmm |
| ST Phases M | Number of ST phases in the past month | Continuous | 0.39% | Pmm |
| ST Phases W | Number of ST phases in the past week | Continuous | 0.39% | Pmm |
| ST Prob. | Probability of ST in the future | Likert [0 – 4] | 0.39% | Pmm |
| Suicide Plan | Suicide Plan in lifetime | Factor [Yes, No] | 0.39% | Logreg |
| Suicidal Gesture | Suicidal Gesture in lifetime | Factor [Yes, No] | 0.78% | Logreg |
| Suicide Attempt | Suicide Attempt in lifetime | Factor [Yes, No] | 0.78% | Logreg |
| NSSI Thoughts | Thoughts of NSSI in lifetime | Factor [Yes, No] | 0% | - |
| NSSI-T Phases L | Number of NSSI-T phases in lifetime | Continuous | 1.57% | Pmm |
| NSSI-T Phases M | Number of NSSI-T phases in the past month | Continuous | 0.39% | Pmm |
| NSSI-T Prob. | Probability of Thoughts of NSSI in the future | Likert [0 – 4] | 0.39% | Pmm |
| NSSI | NSSI in lifetime | Factor [Yes, No] | 0% | - |
| NSSI L | Number of NSSI in lifetime | Continuous | 1.18% | Pmm |
| NSSI M | Number of NSSI in the past month | Continuous | 0.39% | Pmm |
| NSSI Med. Treatment | Medical Treatment because of NSSI in lifetime | Factor [Yes, No] | 0% | - |
| NSSI Prob. | Probability of NSSI in the future | Likert [0 – 4] | 0% | - |
|  |  |  |  |  |
| Clinical Measures |  |  |  |  |
| Medical Treatment | Current medical treatment | Factor [Yes; No] | 0% | - |
| First Contact | Year of first clinical contact | Continuous | 18.82% | Pmm |
| AVPD Symptoms | Number of AVPD Symptoms | Continuous | 1.18% | Pmm |
| BPD Symptoms | Number of BPD Symptoms | Continuous | 0.39% | Pmm |
| CD Symptoms | Number of CD Symptoms | Continuous | 1.18% | Pmm |
| DIKJ | DIKJ Raw Scores | Continuous | 2.75% | Norm |
| DAPP-ED | DAPP-BQ cluster score: Emotional dysregulation | Continuous | 3.14% | Norm |
| DAPP-DB | DAPP-BQ cluster score: Dissocial behavior | Continuous | 3.53% | Norm |
| DAPP-SA | DAPP-BQ cluster score: Social avoidance | Continuous | 7.84% | Norm |
| DAPP-C | DAPP-BQ cluster score: Compulsiveness | Continuous | 2.75% | Norm |
|  |  |  |  |  |
| Global Clinical Impressions |  |  |  |  |
| GAF | Global Assessment of Functioning | Continuous | 20.78% | Norm |
| CGI-S | Clinical Global Impression – Severity | Likert [0 – 7] | 7.06% | Pmm |
| KID-10 | KIDSCREEN-10 Index | T-Values | 10.20% | Norm |
| GSI | Global Severity Index of the SCL-90-R | Continuous | 0.78% | Norm |
|  |  |  |  |  |
| Adverse Experiences |  |  |  |  |
| Sexual Abuse | Victim of sexual abuse in lifetime | Factor [Yes; No] | 2.35% | Logreg |
| Physical Abuse | Victim of physical abuse in lifetime | Factor [Yes; No] | 5.10% | Logreg |
| Neglect | Emotional Neglect from at least one parent | Factor [Yes; No] | 4.31% | Logreg |
| Antipathy | Antipathy from at least one parent | Factor [Yes; No] | 4.31% | Logreg |
|  |  |  |  |  |

Abbreviations: SITBI, self-injurious thoughts and behaviors interview. ST, suicidal thoughts. NSSI, non-suicidal self-injury. AVPD, avoidant personality disorder. BPD, borderline personality disorder. CD, conduct disorder. DIKJ, depression inventory for children and adolescents. DAPP, dimensional assessment of personality pathology.

The predictors were assessed using the following tools:(a) Socio-demographic information, including age, gender, weight, height, and current school type, as well as family characteristics including whether the patient is an only child, lives with their mother and / or father, and the cohabitation status of their parents were assessed in a standardized way. (b) The German version of the *Self-injurious thoughts and behaviors interview* (SITBI-G) (Fischer et al., 2014; Nock et al., 2007) was used to obtain details regarding a patient’s suicidal thoughts and gestures, non-suicidal self-injury (NSSI), suicide plans, previous SA and self-rated risk for future SA and NSSI. The SITBI items assessing the number of ST phases (and analogously the NSSI phases) over different time frames—lifetime, past month, and past week—are not mutually exclusive. Rather, the shorter time spans are nested within the longer ones. That is, any episode reported in the past week is, by definition, also part of the past month and of lifetime history, and any episode in the past month is necessarily part of lifetime history. Thus, the variables partially overlap due to the hierarchical nature of the time windows. (c) Clinical measures included medical treatment of the patient, and the year of first contact to a professional child and adolescent psychiatric service. Psychiatric pathology, including the number of symptoms of avoidant personality disorder (AVPD), borderline personality disorder (BPD), and conduct disorder (CD), were obtained via the *Structured Clinical Interview for DSM-IV, Axis II* (SKID-II) [3] and the *Mini International Neuropsychiatric Interview for Children and Adolescents* (MINI KID) [4]. The *Depression Inventory for Children and Adolescents* (DIKJ) [5] was used to assess the extent of depressive symptoms. The *Dimensional Assessment of Personality Pathology – Basic Questionnaire* (DAPP-BQ) [6], specifically cluster scores for emotional dysregulation, dissocial behavior, social avoidance, and compulsiveness, were used to assess dimensional aspects of personality disorders. (d) Global clinical impressions included overall functioning – determined by the *Global Assessment of Functioning* (GAF) [7], overall psychological distress – determined by the *Symptom Checklist-90-R Global Severity Index* (GSI) [8], severity of illness – determined by the *Clinical Global Impression – Severity* scale (CGI-S) [9], and health-related quality of life – determined by the *KIDSCREEN-10* [10]. (e) Adverse Childhood Experiences (ACE) including sexual abuse, physical abuse, neglect and antipathy were measured using the German version of the *Childhood Experience of Care and Abuse Questionnaire* (CECA.Q) [11].

A correlation matrix of all predictors is displayed in SFigure 1.

**SFigure 1.**
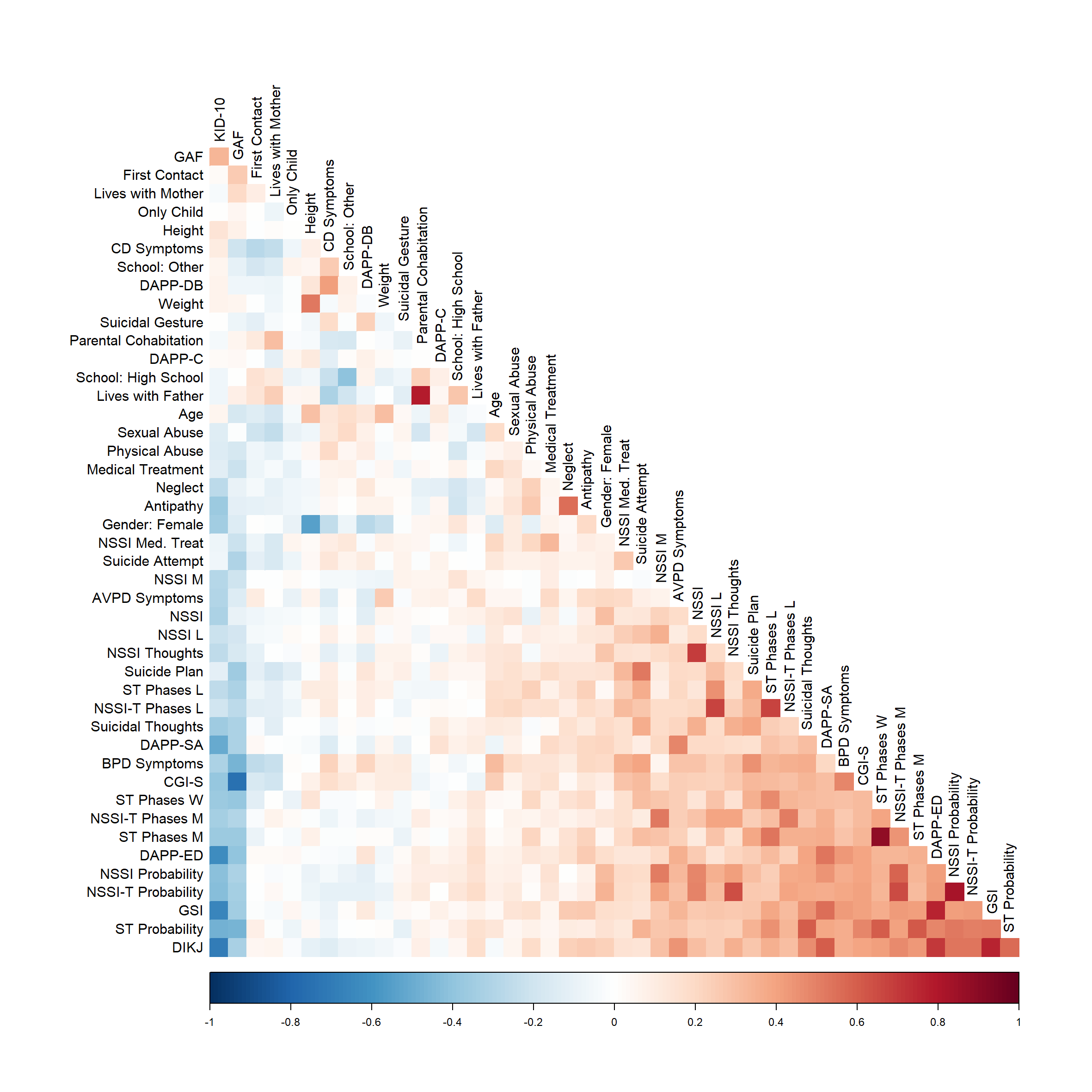
Correlation Matrix of Predictors

*Note:* Pearson correlation computed using all complete variable pairs. Non-numeric variables were one-hot encoded.

*Abbreviations*: GAF, global assessment of functioning. First contact, year of first Contact with mental health care system. CD-Symptoms, conduct disorder symptoms. School: other, currently any other school than secondary or high school. DAPP-DB, dimensional assessment of personality pathology dissocial behavior. DAPP-C, dimensional assessment of personality pathology compulsiveness. Medical Treat., any medical treatment received after non-suicidal self-harm. NSSI M, number of days with non-suicidal self-injury past month. AVPD Symptoms, avoidant personality disorder symptoms. NSSI, any non-suicidal self-injury. NSSI L, number of days with non-suicidal self-injury lifetime. NSSI Thoughts, any thoughts of non-suicidal self-injury. ST Phases L, number of days with suicidal thoughts lifetime. NSSI-T Phases L, number of days with thoughts of non-suicidal self-injury lifetime. DAPP-SA, dimensional assessment of personality pathology social avoidance. BPD Symptoms, borderline personality disorder symptoms. CGI-S, clinical global impression – severity. ST Phases W, number of days with suicidal thoughts past week. NSSI-T Phases M, number of days with thoughts of non-suicidal self-injury past month. ST Phases M, number of days with suicidal thoughts past month. DAPP-ED, dimensional assessment of personality pathology emotional dysregulation. NSSI Probability, self-rated probability of future non-suicidal self-injury. NSSI-T Probability, self-rated probability of future thoughts of non-suicidal self-injury. GSI, global severity index. ST Probability, self-rated possibility of future suicidal thoughts. DIKJ, depression inventory for children and adolescents.

# Missing data

Missing data was imputed via multiple imputation by chained equations[12], using the mice package[13] in R[14]. Predictor variables were missing at various rates, ranging from 0% - 20.78%. In a first step, 500 complete data sets were imputed and used to fit 500 separate models for each algorithm. Standard deviations (SDs) of AUCs were then iteratively aggregated via Rubin’s rules [15, 16], using 2-200 randomly selected imputed data sets. SFigure 2 shows that the SD of AUC-SDs starts to converge at around 60 datasets, indicating that model performance did no longer benefit from adding more imputed data sets to the aggregation process.

**SFigure 2** Change of error in AUC-SD based on number of imputed data sets

*
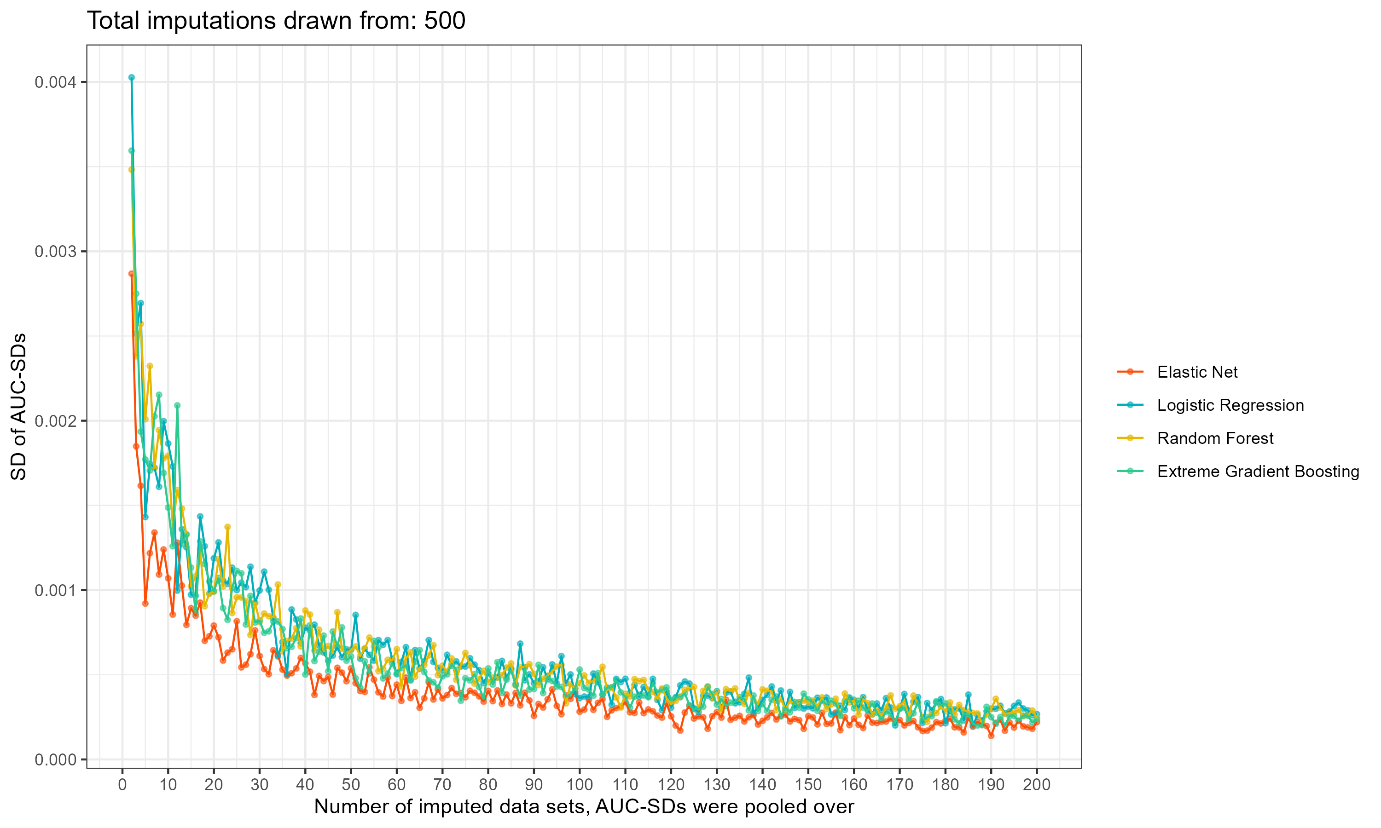
*

*Abbreviations*: AUC = Area under the receiver operator characteristic curve. SD = standard deviation.

Thus, all results from our data analysis were aggregated over the first 60 trained models for each algorithm.

The specific imputation method was based on the variable type of each predictor. Logistic regression (Logreg) was used for binary variables; Polytmous logistic regression (Polyreg) was used for unordered factor variables; Bayesian linear regression (Norm) was used for continuous variables; Predictive mean matching (Pmm) was used for Likert variables and continuous variables, in cases where Norm would produce values outside the possible variable range. STable 1 shows a detailed overview of all predictors including variable type, missing rate and imputation method.

An additional analysis was conducted on all patients who had no missing values (N=139) (details below, STable3).

**Statistical analysis**

All models were trained using the same data. For each algorithm a total of sixty models were individually trained, one for each imputed data set. In each training phase data was split into training and test sets, using five-fold repeated cross-validation with twenty repetitions. For a particular model a grid-search was performed to optimize tuning parameters. A grid-search trains multiple models using slightly different hyperparameters. The model with the best tuned parameters, based on the area under the receiver operator characteristic curve (AUC) was chosen to obtain the final model parameters. The entire training set was then used to fit a final model.

A set of performance metrics was obtained from each model, including AUC, Brier score, sensitivity, specificity, positive predictive value (PPV) and negative predictive value (NPV). AUC quantifies the overall discriminative ability of a predictive model and ranges from 0.5 (random guessing) to 1 (perfect accuracy). Using heuristic categories, AUC values can be considered *bad* (0.5 - 0.6), *sufficient* (0.6 - 0.7), *good* (0.7 - 0.8), *very good* (0.8 - 0.9) or *excellent* (0.9 - 1.0) [17]. The Brier score quantifies the mean squared difference between the predicted probabilities and the actual outcomes, with lower Brier scores indicating more accurate model calibration. Sensitivity (recall) shows the proportion of correctly predicted SA-positive patients among all true SA-positive patients, while specificity shows the proportion of correctly predicted SA-negative patients among all true SA-negative patients. PPV (precision) is the ratio of true SA-positive patients among all predicted SA-positive patients, and NPV represents the ratio of true SA-negative patients among all predicted SA-negative patients. The choice of performance metrics was intended to cover different aspects of diagnostic accuracy.

Predictor importance was derived differently for each algorithm. For elastic net (EN) and logistic regression (LR), the absolute value of the Beta-coefficients was used. The magnitude of the coefficients reflected the predictors’ relative importance in the model, with larger coefficients indicating stronger influence. For random forest (RF), predictor importance was determined by the magnitude of actual impurity reduction (AIR). AIR is based on the reduction of Gini impurity of splits, but is debiased in regards to favoring predictors with more possible split points (e.g. continuous variables) [18]. For extreme gradient boosting (XGB), importance scores were represented by information gain – a measure that quantifies a predictor’s relative contribution to the model, based on the total gain of a predictor’s splits [19]. Predictors with higher information gain are considered more influential. To facilitate comparisons between algorithms, all importance scores were normalized on a uniform 0-100 scale.

Model training was performed in R [20] via the *caret* package [21]. Packages for specific algorithms included *glmnet* for LR and EN [22], *ranger* for RF [23], and *xgboost* for XGB [19].

**Results**

**Baseline characteristics of patients**

The full baseline characteristics of patients is displayed in STable 2.

**STable 2** Baseline Patient Characteristics

| Predictors | SA-positive (*N* = 96)  N (%) / Mean (SD) | SA-negative (*N* = 159)  N (%) / Mean (SD) | Overall (*N* = 255)  N (%) / Mean (SD) |
| --- | --- | --- | --- |
| Socio-demographics |  |  |  |
| Age | 14.65 (1.51) | 15.16 (1.41) | 14.96 (1.47) |
| Weight | 59 (13) | 60 (14) | 60 (14) |
| Height | 166 (7) | 167 (9) | 167 (8) |
| Female Gender | 87 (91%) | 137 (86%) | 224 (88%) |
| Only Child | 16 (17%) | 25 (16%) | 41 (16%) |
| Parental Cohabitation | 46 (48%) | 68 (43%) | 114 (45%) |
| Lives with Mother | 78 (84%) | 128 (85%) | 206 (84%) |
| Lives with Father | 49 (61%) | 78 (55%) | 127 (57%) |
| School |  |  |  |
| Haupt/Realschule | 33 (34%) | 62 (39%) | 95 (37%) |
| Gymnasium | 50 (52%) | 71 (45%) | 121 (48%) |
| Other School | 13 (14%) | 25 (16%) | 38 (15%) |
|  |  |  |  |
| SITBI Measures |  |  |  |
| Suicidal Thoughts | 95 (99%) | 130 (82%) | 225 (88%) |
| ST Phases L | 268 (333) | 160 (263) | 201 (296) |
| ST Phases M | 14 (11) | 8 (10) | 10 (11) |
| ST Phases W | 3.11 (2.80) | 1.77 (2.43) | 2.28 (2.65) |
| ST Prob. | 2.92 (1.09) | 2.06 (1.53) | 2.39 (1.44) |
| Suicide Plan | 68 (72%) | 68 (43%) | 136 (54%) |
| Suicidal Gesture | 15 (16%) | 23 (15%) | 38 (15%) |
| Suicide Attempt | 73 (77%) | 54 (34%) | 127 (50%) |
| NSSI Thoughts | 91 (95%) | 138 (87%) | 229 (90%) |
| NSSI-T Phases L | 330 (368) | 249 (403) | 280 (391) |
| NSSI-T Phases M | 18 (12) | 11 (11) | 14 (12) |
| NSSI-T Prob. | 3.33 (1.21) | 2.58 (1.59) | 2.86 (1.50) |
| NSSI | 92 (96%) | 144 (91%) | 236 (93%) |
| NSSI L | 161 (194) | 128 (230) | 140 (217) |
| NSSI M | 9 (9) | 6 (8) | 7 (9) |
| NSSI Med. Treatment | 21 (22%) | 23 (14%) | 44 (17%) |
| NSSI Prob. | 3.20 (1.23) | 2.25 (1.60) | 2.61 (1.54) |
|  |  |  |  |
| Clinical Measures |  |  |  |
| Medical Treatment | 14 (15%) | 18 (11%) | 32 (13%) |
| First Contact | 2014.1 (3.1) | 2014.6 (2.9) | 2014.4 (3.0) |
| AVPD Symptoms | 1.93 (1.95) | 1.80 (1.82) | 1.85 (1.87) |
| BPD Symptoms | 4.14 (2.10) | 3.37 (2.41) | 3.66 (2.32) |
| CD Symptoms | 0.98 (1.79) | 0.99 (1.78) | 0.98 (1.78) |
| DIKJ | 32 (8) | 28 (10) | 30 (10) |
| DAPP-ED | 391 (52) | 378 (58) | 383 (56) |
| DAPP-DB | 202 (39) | 202 (39) | 202 (39) |
| DAPP-SA | 157 (23) | 147 (27) | 151 (26) |
| DAPP-C | 42 (11) | 43 (11) | 42 (11) |
|  |  |  |  |
| Global Clinical Impressions |  |  |  |
| GAF | 46 (12) | 51 (13) | 50 (12) |
| CGI-S | 5.28 (0.86) | 4.77 (1.06) | 4.96 (1.02) |
| KID-10 | 34 (6) | 36 (7) | 35 (7) |
| GSI | 1.75 (0.67) | 1.52 (0.71) | 1.61 (0.71) |
|  |  |  |  |
| Adverse Childhood Experiences |  |  |  |
| Sexual Abuse | 17 (18%) | 37 (24%) | 54 (22%) |
| Physical Abuse | 22 (24%) | 38 (25%) | 60 (25%) |
| Neglect | 41 (45%) | 60 (39%) | 101 (41%) |
| Antipathy | 51 (56%) | 84 (55%) | 135 (55%) |

# *Abbreviations:* SITBI, self-injurious thoughts and behaviors interview. Suicidal thoughts, any suicidal thoughts in lifetime. ST phases L, number of days with suicidal thoughts lifetime. ST phases M, number of days with suicidal thoughts past month. ST phases W, number of days with suicidal thoughts past week. ST prob., self-rated probability of future suicidal thoughts. NSSI, any non-suicidal self-injury. NSSI-T Phases L, number of days with thoughts of NSSI lifetime. NSSI-T Phases M, number of days with thoughts of NSSI past month. NSSI-T Prob., self-rated probability of future thoughts of NSSI. NSSI-L, number of days with NSSI lifetime. NSSI-M, number of days with NSSI past month. NSSI Med. Treatment, ever received medical treatment because of NSSI. NSSI Prob., self-rated probability of future NSSI. Medical Treatment, current medical treatment. First Contact, year of first contact to any psychiatric services. AVPD, avoidant personality disorder. BPD, borderline personality disorder. CD, conduct disorder. DIKJ, depression inventory for children and adolescents. DAPP, dimensional assessment of personality pathology. DAPP-ED, DAPP emotion dysregulation cluster score. DAPP-DB, DAPP dissocial behavior cluster score. DAPP-SA, DAPP social avoidance cluster score. DAPP-C, DAPP compulsiveness cluster score. GAF, global assessment of functioning. CGI-S, clinical global impression – severity. KID-10, KIDSCREEN-10. GSI, global severity index.

**Model performance**

SFigure 3 displays ROC curves for all four algorithms including confidence intervals.

**SFigure 3**
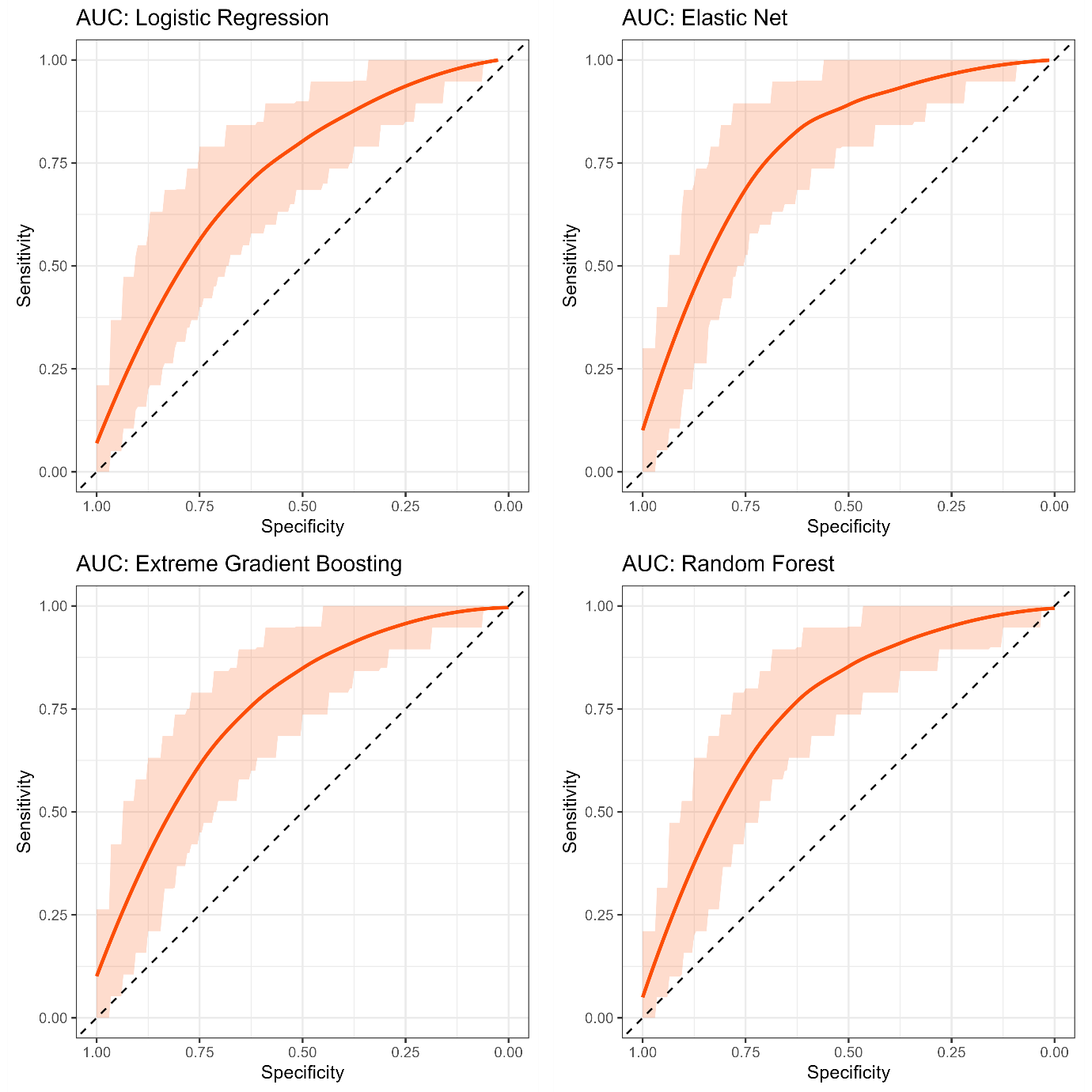
ROC Curves with Confidence Intervals

*Note:* Confidence intervals represent the upper and lower 10^th^ percentile of computed sensitivity values over all folds for each fitted model.

*Abbreviations*: AUC, area under the receiver operator characteristic curve. ROC, receiver operator characteristic.

**Complete Case Analysis**

Results of complete case analysis (N=139) are shown in STable 3 and revealed a similar pattern as the imputed data set.

**STable 3** Complete Case Analysis Results

| Accuracy Measures | LR (SD) | RF (SD) | XGB (SD) | EN (SD) |
| --- | --- | --- | --- | --- |
| Sensitivity | 0.47 (0.16) | 0.47 (0.17) | 0.48 (0.15) | 0.49 (0.16) |
| Specificity | 0.68 (0.12) | 0.85 (0.09) | 0.82 (0.09) | 0.83 (0.09) |
| PPV | 0.45 (0.13) | 0.64 (0.17) | 0.60 (0.14) | 0.62 (0.15) |
| NPV | 0.72 (0.06) | 0.76 (0.06) | 0.76 (0.05) | 0.77 (0.06) |
| AUC | 0.62 (0.11) | 0.76 (0.09) | 0.73 (0.09) | 0.77 (0.09) |
| Brier score | 0.37 (0.08) | 0.19 (0.03) | 0.20 (0.04) | 0.18 (0.03) |

*Note:* The analysis was conducted on all patients (*N* = 139), who had no missing values.

*Abbreviations*: LR, logistic regression. RF, random forest. XGB, extreme gradient boosting. EN, elastic net. SD, standard deviation. PPV, positive predictive value. NPV, negative predictive value. AUC, area under the receiver operator characteristic curve.

**Model calibration and tuning parameters**

A summary of the selected tuning parameters is shown in STable 4.

**STable 4** Model Calibration

| Algorithm | Optimal parameters (SD) |
| --- | --- |
| LR | alpha: 1, lambda: 0 |
| EN | alpha: 1, lambda: 0.04 (0.0004) |
| RF | mtry: 23, min.node.size: 1, splitrule: ‘extratrees’ |
| XGB | eta: 0.3, max_depth: 1, gamma: 0, colsample_bytree: 0.73 (0.1), min_child_weight: 1, subsample: 1, nrounds: 50 |

*Note:* SD represents the standard deviation of parameters for the 60 fitted models, in case the parameters were not identical for all models.

Abbreviations: LR, logistic regression. EN, elastic net. RF, random forest. XGB, extreme gradient boosting.

**Predictor Importance**

The ranks of all predictors are shown in STable 5.

**STable 5** Predictor Importance Rank

| Predictor | EN | LR | RF | XGB |
| --- | --- | --- | --- | --- |
| Suicide Attempt | 1 | 1 | 1 | 1 |
| Age | 2 | 9 | 5 | 2 |
| NSSI Probability | 3 | 6 | 2 | 8 |
| CGI-S | 5 | 5 | 9 | 5 |
| ST Probability | 6 | 24 | 6 | 4 |
| NSSI-T Phases M | 8 | 29 | 3 | 3 |
| Sexual Abuse | 7 | 8 | 23 | 21 |
| Suicide Plan | 10 | 11 | 4 | 34.5 |
| Suicidal Thoughts | 4 | 2 | 24 | 34.5 |
| GAF | 12 | 31 | 17 | 15 |
| DAPP-SA | 11 | 39 | 16 | 13 |
| NSSI M | 29.5 | 40 | 8 | 6 |
| NSSI-T Probability | 29.5 | 13 | 10 | 34.5 |
| First Contact | 9 | 27 | 35 | 19 |
| DAPP-C | 29.5 | 38 | 15 | 9 |
| NSSI-T Phases L | 29.5 | 45 | 13 | 7 |
| ST Phases M | 29.5 | 32 | 11 | 22 |
| ST Phases L | 29.5 | 44 | 12 | 10 |
| ST Phases W | 29.5 | 25 | 7 | 34.5 |
| NSSI | 29.5 | 4 | 33 | 34.5 |
| NSSI L | 13 | 43 | 22 | 23 |
| Height | 29.5 | 35 | 26 | 11 |
| AVPD Symptoms | 29.5 | 21 | 37 | 14 |
| GSI | 29.5 | 18 | 21 | 34.5 |
| NSSI Thoughts | 29.5 | 3 | 36 | 34.5 |
| Lives with Father: No | 29.5 | 7 | 32 | 34.5 |
| DAPP-ED | 29.5 | 42 | 14 | 18 |
| Lives with Mother: No | 29.5 | 20 | 20 | 34.5 |
| School: High School | 29.5 | 12 | 29 | 34.5 |
| DAPP-DB | 29.5 | 41 | 19 | 16 |
| Weight | 29.5 | 37 | 30 | 12 |
| DIKJ | 29.5 | 34 | 28 | 20 |
| CD Symptoms | 29.5 | 30 | 18 | 34.5 |
| KID-10 | 29.5 | 36 | 31 | 17 |
| Medical Treatment | 29.5 | 16 | 34 | 34.5 |
| Neglect | 29.5 | 14 | 38 | 34.5 |
| Suicidal Gesture | 29.5 | 28 | 25 | 34.5 |
| Physical Abuse | 29.5 | 10 | 44 | 34.5 |
| Parental Cohabitation: No | 29.5 | 15 | 41 | 34.5 |
| BPD Symptoms | 29.5 | 33 | 27 | 34.5 |
| Only Child: No | 29.5 | 17 | 43 | 34.5 |
| NSSI Med. Treat.: No | 29.5 | 23 | 39 | 34.5 |
| Gender: Male | 29.5 | 22 | 42 | 34.5 |
| School: Other | 29.5 | 19 | 45 | 34.5 |

Abbreviations: LR, logistic regression. EN, elastic net. RF, random forest. XGB, extreme gradient boosting. NSSI probability, self-rated probability of future non-suicidal self-injury. CGI-S, clinical global impression – severity. ST Probability, self-rated probability of future suicidal thoughts. NSSI-T Phases M, thoughts of non-suicidal self-injury in the past month. GAF, global assessment of functioning. DAPP-SA, dimensional assessment of personality pathology social avoidance. NSSI M, non-suicidal self-injury in the past month. NSSI-T probability, self-rated probability of future thoughts of non-suicidal self-injury. First contact, year of the first contact to any psychiatric services. DAPP-C, dimensional assessment of personality pathology compulsiveness. ST phases M, phases with suicidal thoughts in the past month. NSSI-T phases L, phases with thoughts of non-suicidal self-injury lifetime. ST phases L, suicidal thoughts phases lifetime. ST phases L, number of suicidal thoughts lifetime. ST phases W, number of suicidal thoughts past week. NSSI L, number of non-suicidal self-injury lifetime. NSSI, any non-suicidal self-injury lifetime. AVPD, number of symptoms of avoidant personality disorder. NSSI Thoughts, any thoughts of non-suicidal self-injury in lifetime. GSI, gobal severity index of symptoms. DAPP-ED, dimensional assessment of personality pathology emotional dysregulation. DAPP-DB, dimensional assessment of personality pathology dissocial behavior. DIKJ, total score symptoms of depression. CD symptoms, symptoms of Conduct disorder. KID10, quality of life. Medical treatment, current medical treatment. Suicidal gesture, any suicidal gesture lifetime. BPD Symptoms, borderline personality symptoms. NSSI med. treat.: no, never received medical treatment because of non-suicidal self-injury. School: other, currently any other school than secondary or high school.

References

1. Fischer G, Ameis N, Parzer P, et al (2014) The German version of the self-injurious thoughts and behaviors interview (SITBI-G): a tool to assess non-suicidal self-injury and suicidal behavior disorder. BMC Psychiatry 14:265. https://doi.org/10.1186/s12888-014-0265-0

2. Nock MK, Holmberg EB, Photos VI, Michel BD (2007) Self-Injurious Thoughts and Behaviors Interview: Development, reliability, and validity in an adolescent sample. Psychol Assess 19:309–317. https://doi.org/10.1037/1040-3590.19.3.309

3. Wittchen H-U, Zaudig M, Fydrich T (1997) SKID. Strukturiertes Klinisches Interview für DSM-IV. Achse I und II. Handanweisung

4. Sheehan DV, Sheehan KH, Shytle RD, et al (2010) Reliability and Validity of the Mini International Neuropsychiatric Interview for Children and Adolescents (MINI-KID). J Clin Psychiatry 71:17393. https://doi.org/10.4088/JCP.09m05305whi

5. Stiensmeier-Pelster J, Schürmann M, Duda K (1989) Depressions-Inventar für Kinder und Jugendliche:(DIKJ). Verlag für Psychologie Dr. CJ Hogrefe

6. Livesley WJ, Jackson D (2009) Manual for the dimensional assessment of personality pathology—basic questionnaire. Port Huron MI Sigma

7. Hall RCW (1995) Global Assessment of Functioning. Psychosomatics 36:267–275. https://doi.org/10.1016/S0033-3182(95)71666-8

8. Derogatis LR, Unger R (2010) Symptom Checklist‐90‐Revised. In: Weiner IB, Craighead WE (eds) The Corsini Encyclopedia of Psychology, 1st ed. Wiley, pp 1–2

9. Busner J, Targum SD (2007) The clinical global impressions scale: applying a research tool in clinical practice. Psychiatry Edgmont 4:28

10. Ravens-Sieberer U, Erhart M, Rajmil L, et al (2010) Reliability, construct and criterion validity of the KIDSCREEN-10 score: a short measure for children and adolescents’ well-being and health-related quality of life. Qual Life Res 19:1487–1500. https://doi.org/10.1007/s11136-010-9706-5

11. Kaess M, Parzer P, Mattern M, et al (2011) Childhood Experiences of Care and Abuse (CECA): Validierung der deutschen Version von Fragebogen und korrespondierendem Interview sowie Ergebnisse einer Untersuchung von Zusammenhängen belastender Kindheitserlebnisse mit suizidalen Verhaltensweisen. Z Für Kinder- Jugendpsychiatrie Psychother 39:243–252. https://doi.org/10.1024/1422-4917/a000115

12. White IR, Royston P, Wood AM (2011) Multiple imputation using chained equations: Issues and guidance for practice. Stat Med 30:377–399. https://doi.org/10.1002/sim.4067

13. Buuren S van, Groothuis-Oudshoorn K (2011) mice: Multivariate Imputation by Chained Equations in R. J Stat Softw 45:1–67. https://doi.org/10.18637/jss.v045.i03

14. R Core Team (2023) R: A Language and Environment for Statistical Computing. R Foundation for Statistical Computing.

15. Eekhout I, van de Wiel MA, Heymans MW (2017) Methods for significance testing of categorical covariates in logistic regression models after multiple imputation: power and applicability analysis. BMC Med Res Methodol 17:129. https://doi.org/10.1186/s12874-017-0404-7

16. Rubin DB (1976) Inference and missing data. Biometrika 63:581–592. https://doi.org/10.1093/biomet/63.3.581

17. Šimundić A-M (2009) Measures of Diagnostic Accuracy: Basic Definitions. EJIFCC 19:203–211

18. Nembrini S, König IR, Wright MN (2018) The revival of the Gini importance? Bioinformatics 34:3711–3718. https://doi.org/10.1093/bioinformatics/bty373

19. Chen T, He T, Benesty M, et al (2023) xgboost: Extreme Gradient Boosting

20. R Core Team (2023) R: A Language and Environment for Statistical Computing. R Foundation for Statistical Computing, Vienna, Austria

21. Kuhn M (2008) Building Predictive Models in *R* Using the **caret** Package. J Stat Softw 28:. https://doi.org/10.18637/jss.v028.i05

22. Friedman J, Hastie T, Tibshirani R (2010) Regularization Paths for Generalized Linear Models via Coordinate Descent. J Stat Softw 33:. https://doi.org/10.18637/jss.v033.i01

23. Wright MN, Ziegler A (2017) **ranger** : A Fast Implementation of Random Forests for High Dimensional Data in *C++* and *R*. J Stat Softw 77:. https://doi.org/10.18637/jss.v077.i01
